# OPTIMIZING CARE TRANSITIONS FOR OLDER ADULTS: A COLLABORATIVE APPROACH THROUGH INTERPROFESSIONAL EDUCATION

**DOI:** 10.1093/geroni/igae098.1425

**Published:** 2024-12-31

**Authors:** Denise Dews

**Affiliations:** University of North Carolina, Chapel Hill, North Carolina, United States

## Abstract

Optimizing care transitions for older adults is a critical aspect of healthcare delivery, particularly given the complex needs and potential social vulnerabilities of this population. Transitioning between different healthcare settings, such as hospitals, rehabilitation facilities, and home care, can be challenging and increase the risk of adverse events for older adults. Interprofessional Education (IPE) offers a collaborative approach to addressing these challenges and improving care transitions for older adults. IPE involves bringing together students and professionals from multiple healthcare disciplines to learn with, from, and about each other to improve collaboration and the quality of patient care. By fostering teamwork, communication, and mutual respect among healthcare providers, IPE can enhance the coordination and continuity of care for older adults during transitions between different care settings. Key strategies for optimizing care transitions for older adults through a collaborative approach with IPE include a team-based approach, comprehensive assessment, care coordination, communication and information sharing, patient and caregiver education, and follow-up and support. By adopting a collaborative approach through IPE, healthcare providers can enhance the quality, safety, and effectiveness of care transitions for older adults, ultimately improving health outcomes and quality of life for this vulnerable population. This presentation will offer strategies for adopting a collaborative approach through Interprofessional Education, thus promoting quality, safety, and effectiveness of care transitions for older adults, ultimately improving health outcomes and quality of life for this vulnerable population.

